# Preparation of Polymerized High Internal Phase Emulsion Membranes with High Open-Cellular Extent and High Toughness via RAFT Polymerization

**DOI:** 10.3390/polym17040515

**Published:** 2025-02-17

**Authors:** Yulan Wu, Jie Huang, Zanru Guo, Qian Yang, Chunmiao Xia, Zhenan Zheng

**Affiliations:** 1 Fujian Provincial Key Laboratory of Modern Analytical Science and Separation Technology, College of Chemistry, Chemical Engineering and Environment, Minnan Normal University, Zhangzhou 363000, China; 2 Anhui Laboratory of Clean Energy Materials and Chemistry for Sustainable Conversion of Natural Resources, School of Chemical and Environmental Engineering, Anhui Polytechnic University, Wuhu 241000, China

**Keywords:** polyHIPE membranes, RAFT polymerization, open-cellular extent, toughness

## Abstract

Porous polymer membranes with highly interconnected open-cellular structure and high toughness are crucial for various application fields. Polymerized high internal phase emulsions (polyHIPEs), which usually exist as monoliths, possess the advantages of high porosity and good connectivity. However, it is difficult to prepare membranes due to brittleness and easy pulverization. Copolymerizing acrylate soft monomers can effectively improve the toughness of polyHIPEs, but it is easy to cause emulsion instability and pore collapse. In this paper, stable HIPEs with a high content of butyl acrylate (41.7 mol% to 75 mol% based on monomers) can be obtained by using a composite emulsifier (30 wt.% based on monomers) consisting of Span80/DDBSS (9/2 in molar ratio) and adding 0.12 mol·L^−1^ CaCl_2_ according to aqueous phase concentration. On this basis, polyHIPE membranes with high open-cellular extent and high toughness are firstly prepared via reversible addition–fragmentation chain transfer (RAFT) polymerization. The addition of the RAFT agent significantly improves the mechanical properties of polyHIPE membranes without affecting open-cellular structure. The toughness of polyHIPE membranes prepared by RAFT polymerization is significantly enhanced compared with conventional free radical polymerization. When the molar ratio of butyl acrylate/styrene/divinylbenzene is 7/4/1, the polyHIPE membrane prepared by RAFT polymerization presents plastic deformation during the tensile test. The toughness modulus reaches 93.04 ± 12.28 kJ·m^−3^ while the open-cellular extent reaches 92.35%, and it also has excellent thermal stability.

## 1. Introduction

Porous polymer membranes play a key role in various application fields, such as separation analysis [1,2,3], sensors [4,5], energy storage and conversion, etc. [6,7,8,9]. An ideal porous polymer membrane should possess a highly interconnected open-cellular structure and high toughness. The highly interconnected open-cellular structure is beneficial to achieving high mass transfer flux with low resistance, while high toughness helps to maintain structure integrality when subjected to external force. The preparation method of porous polymer materials usually includes foaming, phase separation, pore-forming agent, etc. [10,11,12]. However, these methods lack the regulation of the pore structure of materials.

By contrast, polymerized high internal phase emulsions (polyHIPEs) provide a facile method to build open-cellular structures with high porosity and good pore connectivity. Typical polyHIPEs are monoliths with a high glass transition temperature (T_g_), which are produced by free radical crosslinking copolymerization of monomers such as styrene (St) and divinylbenzene (DVB). Although having a high modulus and strength, typical polyHIPEs are extremely brittle, thus easily leading to fragmentation during the preparation of membranes. The mechanical properties of typical polyHIPEs can only be measured by a compression test instead of a tensile test [13]. Therefore, improving the toughness of polyHIPEs is critical in order to expand their application fields [13,14]. In order to address the above problems, Kovačič et al. [13,15,16,17] prepared polyHIPE membranes with improved toughness by ring-opening metathesis polymerization (ROMP) of dicyclopentadiene (DCPD). However, ROMP of DCPD requires strict control of reaction conditions, such as temperature, pressure, and solvent selection, to avoid the occurrence of side reactions and the instability of the product structure [15,16].

Free radical polymerization is a more convenient process due to the wide range of applied monomers and mild reaction conditions. But to prepare polyHIPE membranes with high open-cellular extent and high toughness via free radical polymerization is still challenging. By copolymerizing acrylate soft monomers, the T_g_ of the polymer can be significantly reduced, thereby improving the toughness of polyHIPEs [18]. However, due to the high polarity of the acrylate monomer, Ostwald ripening is overwhelming when its dosage is large, which is detrimental to the formation of a stable emulsion [19]. Furthermore, copolymerizing too many soft monomers will lead to the collapse and closure of pores, affecting the formation of an open-cellular structure [20]. To date, the concerned research is limited, and there is a lack of discussion on the intrinsic structure and mechanical properties of the materials.

Previous studies have shown that controlled/“living” free radical polymerization (CLRP) realizes the controllability of the polymerization process by changing the kinetic characteristics, thereby obtaining a more uniform crosslinking structure and further improving the mechanical properties of the material [21,22,23,24,25,26]. This provides a new direction for the preparation of polyHIPE membranes with high open-cellular extent and high toughness. In this paper, polyHIPE membranes composed of monomers, including styrene (St), butyl acrylate (BA), and divinylbenzene (DVB), composite emulsifiers including sorbitol monooleate (Span 80) and sodium dodecyl benzene sulfonate (DDBSS), are firstly prepared via reversible addition–fragmentation chain transfer (RAFT) polymerization, one of the controlled/“living” free radical polymerizations. The effects of RAFT polymerization and monomer composition on the intrinsic structure and mechanical properties of polyHIPE membranes are systematically investigated in order to achieve high open-cellular extent and high toughness.

## 2. Experimental Section

### 2.1. Materials

**Figure 1:**
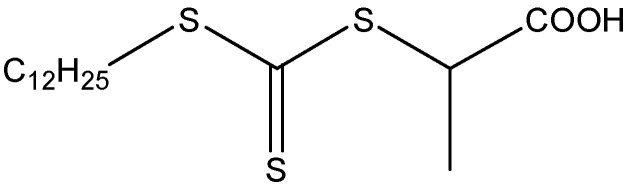
The molecular formula of RAFT agent.

In order to remove the inhibitor, styrene (St, M_n_ = 104.15 g·mol^−1^, Xilong Scientific Co., Ltd., Shantou, Guangdong, China) was distilled under reduced pressure, and butyl acrylate (BA, M_n_ = 128.19 g·mol^−1^, Xilong Scientific Co., Ltd., Shantou, Guangdong, China) was washed with a 10 wt.% sodium hydroxide solution for more than three times. Divinylbenzene (DVB, M_n_ = 130.19 g·mol^−1^, 80%, Sigma-Aldrich, St. Louis, MO, USA), 2,2′-azobis(2-methylpropionitrile) (AIBN, M_n_ = 164.21 g·mol^−1^, 99%, Shanghai Aladdin Biochemical Technology Co., Ltd., Shanghai, China), sorbitan oleate (Span 80, M_n_ = 428.61 g·mol^−1^, Xilong Scientific Co., Ltd., Shantou, Guangdong, China), sodium dodecyl benzene sulfonate (DDBSS, M_n_ = 348.48 g·mol^−1^, 90%, Macklin, Shanghai, China), and calcium chloride (CaCl_2_, M_n_ = 110.98 g·mol^−1^, anhydrous, Xilong Scientific Co., Ltd., Shantou, Guangdong, China) were used as purchased without further purification. The 2-{[(Dodecyl-sulfanyl) carbonothioyl] sulfanyl} propanoic acid (M_n_ = 350 g·mol^−1^) was synthesized and purified as described in reference [27], which was used as the RAFT agent. The molecular formula of the RAFT agent is shown in Figure 1.

### 2.2. Preparation of polyHIPE Membranes

Take RAFT-2 as an example: the organic phase was firstly prepared by mixing 4.480 g BA (3.5 × 10^−2^ mol), 0.809 g St (7.77 × 10^−3^ mol), 0.506 g DVB (3.89 × 10^−3^ mol), and 0.0652 g the RAFT agent (1.86 × 10^−4^ mol), 1.472 g Span 80 (3.43 × 10^−3^ mol) and 0.0513 g AIBN (3.12 × 10^−4^ mol). Then, it was poured into a three-necked round-bottom flask. An amount of 0.266 g CaCl_2_ (2.40 × 10^−3^ mol), 0.266 g DDBSS (7.63 × 10^−4^ mol) was dissolved in 20 g of deionized water to form the aqueous phase. The aqueous phase was then dropwise added to the organic phase in the flask while stirring at 300 rpm. Once the addition was completed, the stirring speed was adjusted to 1600 rpm and continued for 30 min to obtain a viscous, homogeneous emulsion. The emulsion was poured into a mold and transferred to a 70 °C drying oven for polymerization. After 72 h, the samples were removed from the mold and placed in a Soxhlet extractor. They were first extracted with deionized water for 24 h, followed by anhydrous ethanol for another 24 h. After extraction, the samples were vacuum dried at 70 °C for 10 h, and polyHIPE membranes were finally obtained.

### 2.3. Characterization

Pore structure. The scanning electron microscope (JSM-6010LA, JEOL Ltd., Tokyo, Japan) was applied to observe the internal structure of polyHIPE membranes. The membrane surface was sputtered for 120 s in a vacuum before observation, and the test voltage was 10 kV.

Specific surface area. The surface area and pore size analyzer (Tristar II 3020, Micromeritics, Norcross, GA, USA) was applied to measure the specific surface area of polyHIPE membranes. The temperature of nitrogen adsorption was set as −196 °C, and the degassing temperature was 100 °C.

Porosity. The mercury porosimeter (Auto Pore IV 9510, Micromeritics, Norcross, GA, USA) was applied to measure the porosity of polyHIPE membranes.

Tensile properties. The tensile properties of polyHIPE membranes were measured using a Zwick/Roell Z020 (ZwickRoell, Ulm, Germany). The shape of the test bar conformed to GB/T 1040.2-2006 [28]. The testing was conducted at 15 °C with a tensile speed of 1 mm·min^−1^. The tensile test was repeated at least three times. The modulus of toughness (M_T_) was calculated using Equation (1) as follows:

(1) $$M_{T}=\int_{0}^{\varepsilon_{f}}\sigma d\varepsilon$$

where σ is tensile strength, ε is strain, and ε_f_ is elongation at break.

Heat stability. The heat stability of polyHIPE membranes was measured using a Pyris 1 TGA (Perkin-Elmer, Ulm, Germany) in a nitrogen atmosphere. The range of test temperature was 30 °C to 700 °C with a heat speed of 10 °C·min^−1^.

Glass transition temperature. A TA Q200 differential scanning calorimeter (TA Instruments, New Castle, DE, USA) was applied to measure the T_g_ of polyHIPE membrane. The test was conducted in a nitrogen atmosphere, and the test temperature range was −40 °C to 100 °C with a heat speed of 10 °C·min^−1^.

## 3. Results and Discussion

The introduction of polar monomers into the HIPE is conducive to Ostwald ripening, resulting in emulsion instability and thus affects the structure and properties of polyHIPEs [29]. Previous studies have shown that Ostwald ripening can be effectively inhibited by introducing electrolyte [30] and a composite emulsifier [31] composed of nonionic and anionic emulsifiers. In this paper, a composite emulsifier (Span80/DDBSS equal to 9/2 in molar ratio) and an electrolyte (0.12 mol·L^−1^ CaCl_2_) were applied to enhance the emulsion stability. The amount of composite emulsifier was 30 wt.% of the total monomers. The synthesized recipes and the corresponding emulsion stability are summarized in Table 1. It can be seen that emulsions with a BA content less than 75 mol% remain stable at 25 °C for more than 15 days, indicating superior stability. However, when the BA content reaches 92 mol%, the emulsion becomes unstable after one day, which is attributed to serious Ostwald ripening.

**Figure 2:**
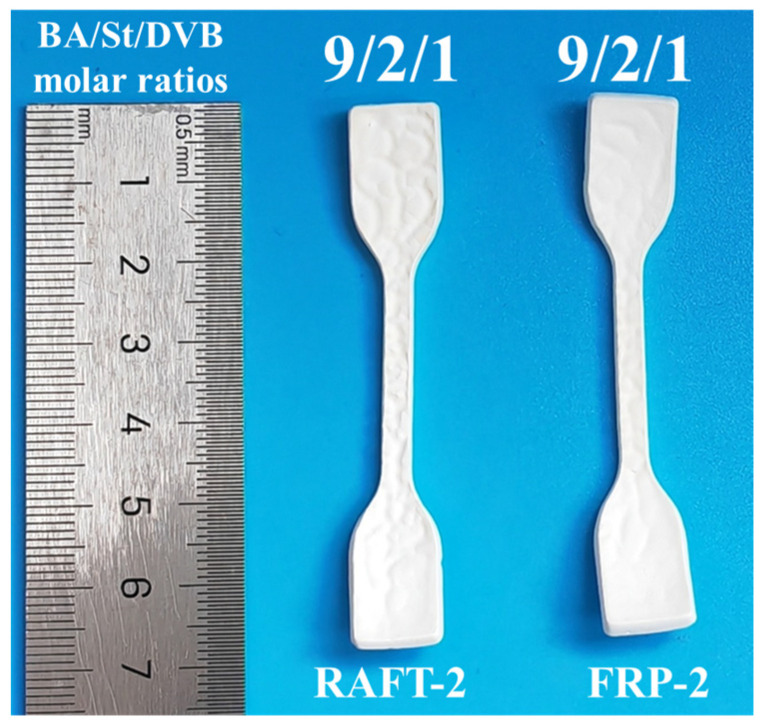
The optical images of polyHIPE membranes prepared by FRP and RAFT polymerization.

**Figure 3:**
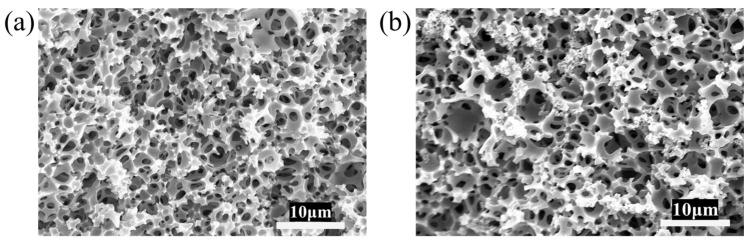
SEM images of polyHIPE membranes prepared by FRP and RAFT polymerization: (**a**) RAFT-2, (**b**) FRP-2.

The stable HIPEs were poured into the mold followed by thermally initiated polymerization to prepare polyHIPE membranes. The effect of conventional free radical polymerization (FRP) and RAFT polymerization on the structure and properties of polyHIPE membranes are compared while maintaining the molar ratio of BA/St/DVB equal to 9/2/1. As shown in Figure 2, the size of the samples is basically the same and the appearance is white, indicating that there is an obvious phase separation structure inside the samples. As presented in Figure 3, two kinds of polyHIPE membranes both exhibit an interconnecting open-cellular structure. Herein, the pore connectivity rate ($P_{C}$) is defined to characterize the open-cellular extent inside polyHIPE membranes, which can be calculated by Equation (2):

(2) $$P_{C}{=P}_{A}/P_{T}$$

where P_A_ is the actual porosity measured by a mercury porosimeter and P_T_ is the theoretical porosity calculated by Equation (3):

(3) PT=VH2OVH2O+VSt+VDVB+VBA=mH2OρH2OmH2OρH2O+mStρSt+mDVBρDVB+mBAρBA

where the $V_{H_{2}O}$ is the volume of water phase, $V_{St},V_{DVB},V_{BA}$ are the volumes of different ingredients, $m_{St},m_{DVB},m_{BA}$ are the actual weights of different ingredients, and $\rho_{H_{2}O},\rho_{St},\rho_{DVB},\rho_{BA}$ are the densities of H_2_O, St, DVB and BA, which are 1, 0.902, 0.919 and 0.898 g·cm^−3^, respectively [32].

**Figure 4:**
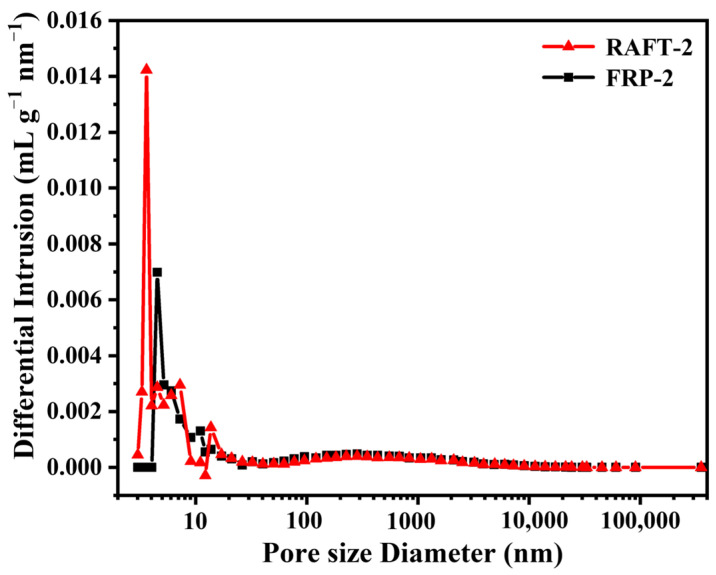
The pore size distribution curves of polyHIPE membranes prepared by FRP and RAFT polymerization.

**Figure 5:**
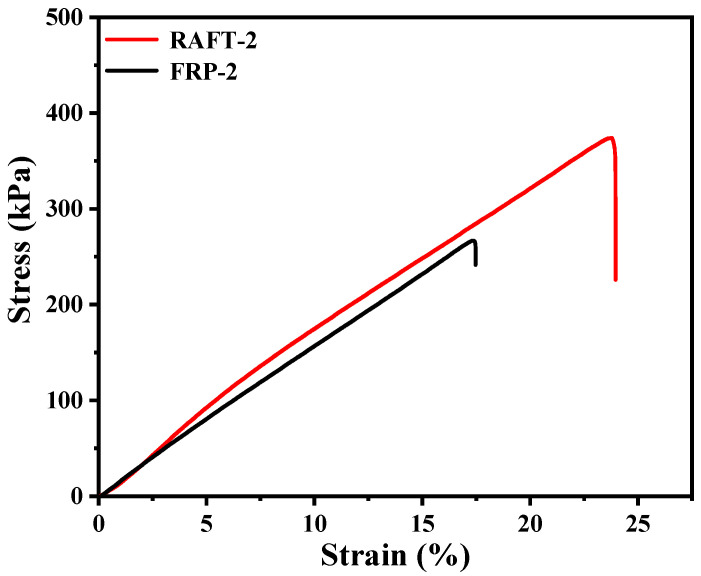
The stress–strain curves of polyHIPE membranes prepared by FRP and RAFT polymerization.

The results of mercury porosimeter are summarized in Table 2. As can be seen, the specific surface area and pore connectivity of RAFT-2 and FRP-2 are basically consistent. However, the Pc of RAFT-2 and FRP-2 is merely around 90%. It is speculated that the high proportion of the soft monomer leads to the insufficient mechanical strength and thus results in partial pore collapse and closure [20,33], which will be elaborated in the following discussion. As shown in the pore size distribution curves (Figure 4), RAFT-2 contains a large amount of smaller mesopores with a size about 1–2 nm, which does not appear in FRP-2, indicating remarkable difference in microstructure for materials prepared by RAFT polymerization and conventional free radical polymerization [34]. The stress–strain curves of synthesized polyHIPE membranes are shown in Figure 5 with results summarized in Table 2. As can be seen, the tensile strength, elongation at break, and modulus of toughness of RAFT-2 are obviously higher than those of FRP-2. Specifically, the tensile strength enhances from 266.8 ± 7.05 kPa to 347 ± 4.31 kPa, and elongation at break increases from 17.36 ± 2.40% to 24.00 ± 3.26%. Correspondingly, the M_T_ of RAFT-2 reaches to 47.60 ± 7.88 kJ·m^−3^, which is about twice that of FRP-2.

**Figure 6:**
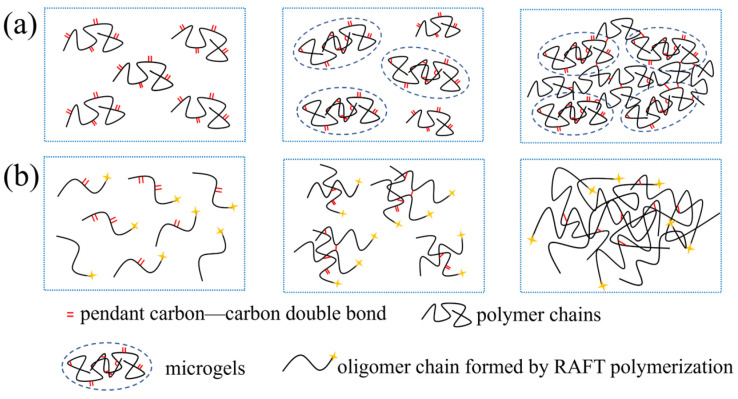
Illustration of the crosslinking process of (**a**) conventional free radical polymerization and (**b**) RAFT polymerization.

The difference in the structure and properties of polyHIPE membranes prepared by RAFT polymerization and conventional free radical polymerization are intimately related to the kinetic characteristics of the polymerization process. In conventional free radical polymerization, the radicals formed by the decomposition of the initiator rapidly grow into polymer chains with high molecular weight, and then terminate in a very short time. Due to the low concentration of polymer chains, cyclization and intramolecular crosslinking reactions are prone to form microgels. As the reaction proceeds, the microgels form a network structure through intermolecular crosslinking [35], as illustrated in Figure 6a. The crosslinking network formed by microgels is heterogeneous and contains a lot of structural defects, which is unfavorable to the mechanical properties. By contrast, all of the polymer chain are generated at the beginning and grow simultaneously during RAFT polymerization. Therefore, a large number of the oligomer living chains tend to form a uniform intermolecular crosslinking network with less structural defects, as illustrated in Figure 6b, which helps to improve the mechanical properties [36].

**Figure 7:**
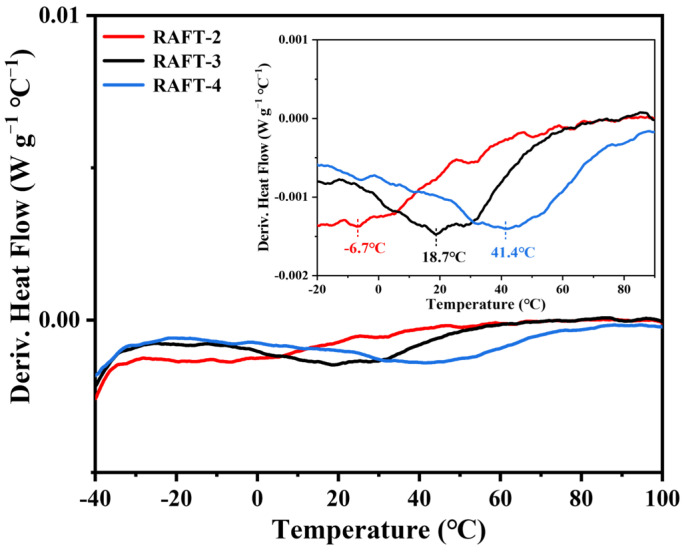
The derivative DSC curves of RAFT-2, RAFT-3, and RAFT-4.

**Figure 8:**
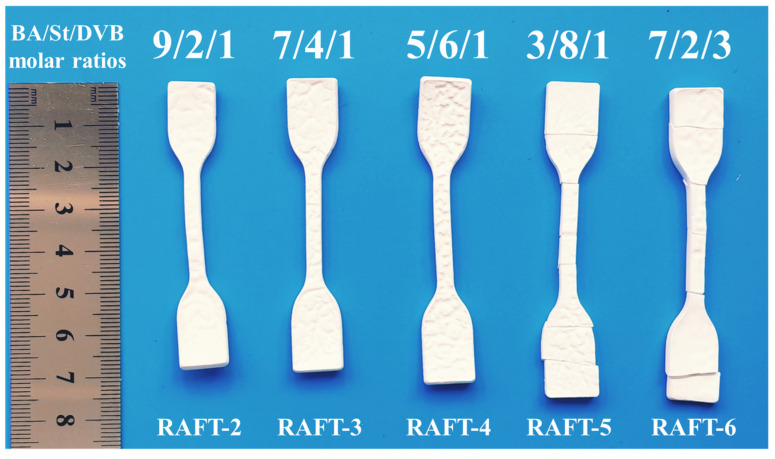
The optical images of polyHIPE membranes obtained by different molar ratios of BA/St/DVB prepared by RAFT polymerization.

**Figure 9:**
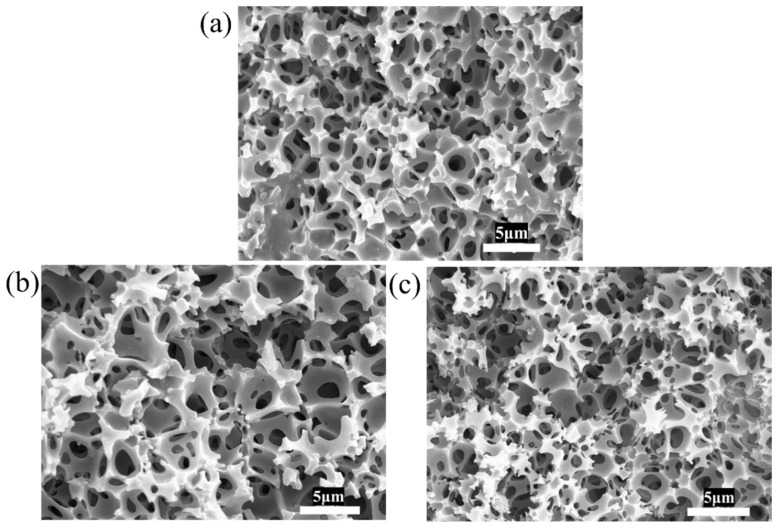
SEM images of polyHIPE membranes with different molar ratios of BA/St/DVB prepared by RAFT polymerization: (**a**) 9/2/1, (**b**) 7/4/1, and (**c**) 5/6/1.

Although RAFT polymerization effectively improves the toughness of polyHIPE membranes, the DSC results show that the T_g_ of RAFT-2 is about −6.7 °C (Figure 7), which is lower than test temperature (i.e., 15 °C), indicating that RAFT-2 is in a rubbery state, so Young’s modulus and the tensile strength of RAFT-2 are still low. The insufficient mechanical strength leads to incomplete open-cellular structures, as described above. Aiming at the above problems, the strength of the material is enhanced by increasing the molar ratio of DVB or St to obtain higher toughness and open-cellular extent. Five kinds of polyHIPE membranes with different monomer ratios based on stable emulsions are synthesized via RAFT polymerization, as listed in Table 1. When the molar ratio of BA/St/DVB changes from 9/2/1 to 7/2/3, RAFT-6 shows brittle fracture, as shown in Figure 8, indicating that the amount of DVB has a significant effect on the brittleness of the material. By changing the molar ratio of BA/St/DVB from 9/2/1 to 5/6/1, RAFT-3 and RAFT-4 remain intact and the degree of shrinkage is reduced. However, when the molar ratio of BA/St/DVB changes to 3/8/1 in RAFT-5, brittle fracture is observed again, as shown in Figure 8. The SEM image (Figure 9) shows that all polyHIPE membranes have an interconnected open-cellular structure when the molar ratio of BA/St/DVB changes from 9/2/1 to 5/6/1. The changes of molar ratios of BA/St/DVB also cause the P_C_ to increase from 88.80% to 100%, which is consistent with the change of sample size, indicating that the collapse and closure of pores can be avoided effectively by increasing the St content. The specific surface area increases from 3.42 m^2^·g^−1^ to 5.19 m^2^·g^−1^, as shown in Table 3.

**Figure 10:**
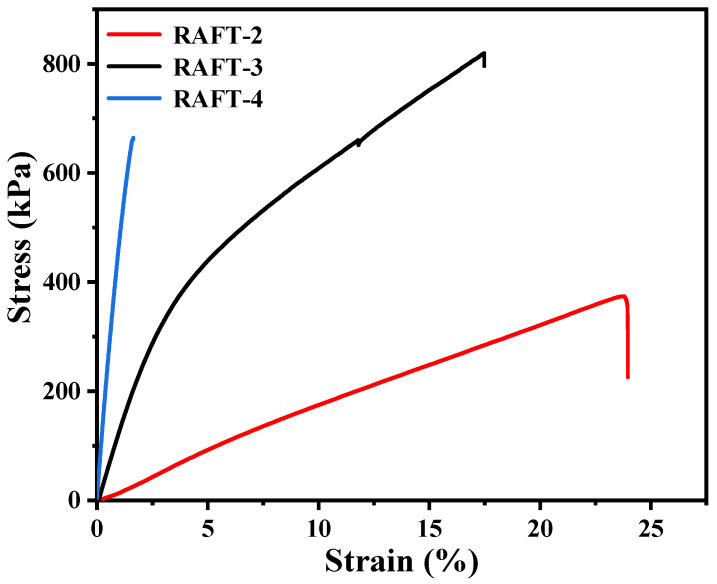
The stress–strain curves of polyHIPE membranes with different molar ratios of BA/St/DVB prepared by RAFT polymerization.

**Figure 11:**
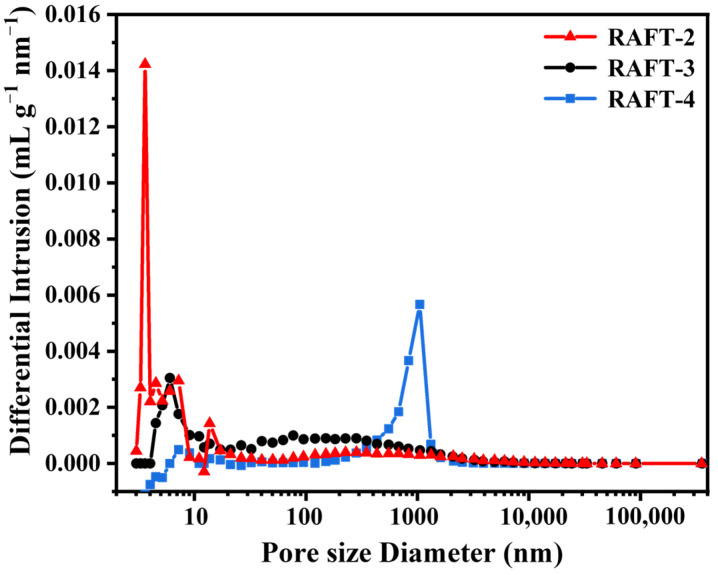
Pore size distribution curves of polyHIPE membranes with different molar ratios of BA/St/DVB prepared by RAFT polymerization.

A tensile test of polyHIPE membranes with different molar ratios of BA/St/DVB were further conducted. The test results are summarized in Table 3, while the stress–strain curves are shown in Figure 10. When the molar ratio of BA/St/DVB changes from 9/2/1 to 7/4/1, the T_g_ of RAFT-3 increases to 18.7 °C (Figure 7), and a distinct yield point is observed in the stress–strain curve. Because the test temperature is close to the T_g_, the yield stress of RAFT-3 is lower than the fracture stress. The frozen chain segment of the glassy RAFT-3 begins to move under external force after the yield point, and the extension of the polymer chain provides significant deformation of the material. Therefore, RAFT-3 exhibits plastic deformation with ductile fracture. Specifically, although elongation at break decreases from 24.00 ± 3.26% to 17.50 ± 3.90%, Young’s modulus and the tensile strength increase to 10.09 ± 1.82 MPa and 819.62 ± 33.31 kPa, respectively. Accordingly, the M_T_ increases from 47.60 ± 7.88 kJ·m^−3^ to 93.04 ± 12.28 kJ·m^−3^. When the molar ratio of BA/St/DVB changes to 5/6/1, the T_g_ of RAFT-4 increases to 41.7 °C (Figure 7) and Young’s modulus of RAFT-4 increases sharply, indicating that the strength of the polymer skeleton is greatly enhanced. Simultaneously, a large number of macropores with a size of several hundred nanometers to microns appear inside the material, while the mesopores with a size below 10 nm basically disappear, as shown in Figure 11, which is consistent with the research of Luo et al. [36]. The results indicate that the existence of mesopores inside RAFT-2 and RAFT-3 with high BA content may be attributed to the collapse of pores caused by insufficient polymer skeleton strength, rather than structural defects caused by uneven crosslinking. However, because the test temperature is much lower than the T_g_, the yield stress of RAFT-4 is higher than the fracture stress. Thus, elongation at break and toughness of RAFT-4 is sharply reduced, and RAFT-4 responds in an almost linear–elastic manner and fails by brittle fracture. Therefore, the adjustment range of the molar ratios of BA/St/DVB is quite narrow in order to achieve high open-cellular extent and high toughness simultaneously in polyHIPE membranes.

**Figure 12:**
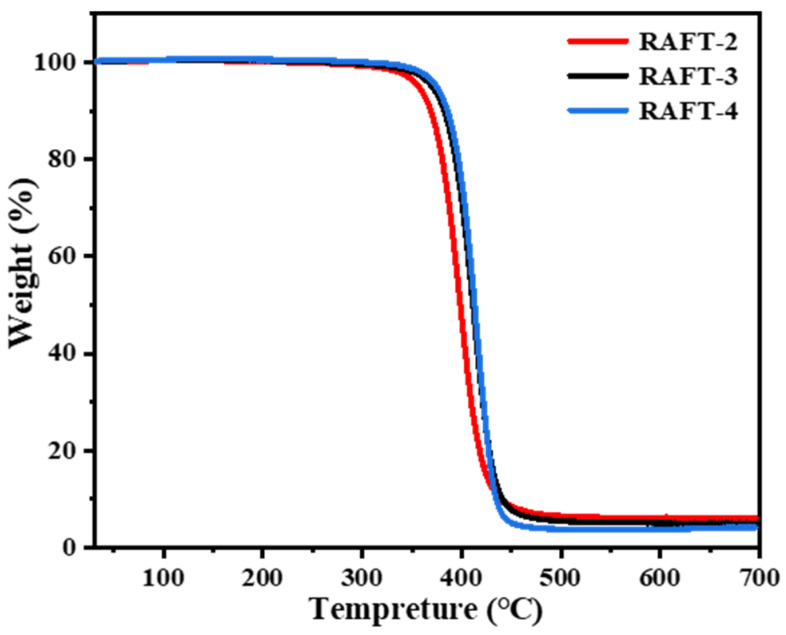
Thermogravimetry analysis curves of RAFT-2, RAFT-3, and RAFT-4.

**Figure 13:**
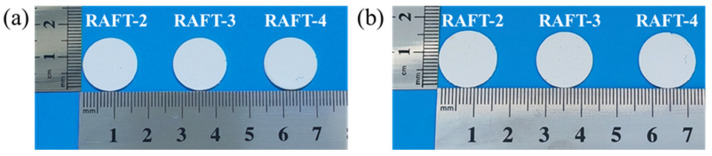
Optical photographs of RAFT-2, RAFT-3 and RAFT-4: (**a**) before and (**b**) after exposure at 200 °C for 30 min.

The heat stability of RAFT-2, RAFT-3, and RAFT-4 was also analyzed. The TGA results show that the initial decomposition temperatures of the samples all exceed 300 °C, and the heat resistance is improved by increasing the St content, as shown in Figure 12. Moreover, no shrinkage or yellowing phenomenon is observed after 200 °C treatment in air for 30 min, as shown in Figure 13, proving excellent thermal stability of all the samples. The synthesized polyHIPE membranes have wide application prospect in the field of lithium ion batteries as separators due to their high open-cellular structures and high toughness. Since the traditional polyolefin separators possesses poor heat stability, the superior heat stability of the polyHIPE membrane can effectively delay the thermal runaway and thus improve the safety of lithium ion batteries.

## 4. Conclusions

In this paper, stable HIPEs with a high butyl acrylate content (41.7 mol% to 75 mol% based on monomers) can be obtained by using a composite emulsifier (30 wt.% based on monomer) consisting of Span80/DDBSS (9/2 in molar ratio) and adding 0.12 mol·L^−1^ CaCl_2_ according to aqueous phase concentration. On this basis, polyHIPE membranes with high open-cellular extent and high toughness are firstly prepared by RAFT polymerization. RAFT polymerization can significantly improve the toughness of the material. When the molar ratio of BA/St/DVB equals 9/2/1, the M_T_ of the polyHIPE membrane prepared by RAFT polymerization is twofold that of traditional free radical polymerization, reaching 47.6 ± 7.88 kJ·m^−3^, while the P_C_ almost remains the same. When the molar ratio of BA/St/DVB equals 7/4/1, the polyHIPE membrane prepared by RAFT polymerization shows plastic deformation during the tensile test, and the M_T_ is further improved to 93.04 ± 12.28 kJ·m^−3^ with a P_C_ of 92.35%, and it also exhibits excellent thermal stability. This work provides a new idea to prepare polyHIPE membranes with high open-cellular structures and high toughness, and thus expands the application field of polyHIPEs.

## Figures and Tables

**Table 1:** The recipe and stability of HIPEs.

Sample	BA/St/DVB ^a^	RAFT ^b^ (mol%)	Emulsifier (30 wt.%)	Electrolyte (mol·L^−1^)	Initiator ^b^ (mol%)	O/W ^c^	Emulsion Stability (days)
			Span80/DDBSS	CaCl_2_	AIBN		
RAFT-1	11/0/1	0.4	9/2	0.12	0.67	1/3	>1
FRP-2	9/2/1	—					>15
RAFT-2	9/2/1	0.4					>15
RAFT-3	7/4/1	0.4					>15
RAFT-4	5/6/1	0.4					>15
RAFT-5	3/8/1	0.4					>15
RAFT-6	7/2/3	0.4					>15

^a^ molar ratio, ^b^ molar ratio of monomers, ^c^ volume ratio.

**Table 2:** The structure and mechanical properties of polyHIPE membranes prepared by FRP and RAFT polymerization.

Sample	P_A_ (%)	P_T_ (%)	P_C_ (%)	BET (m^2^·g^−1^)	E ^a^ (MPa)	σ ^b^ (kPa)	ε_f_ ^c^ (%)	M_T_ ^d^ (kJ·m^−3^)
RAFT-2	67.21	75.69	88.80	3.42	2.04 ± 0.34	347 ± 4.31	24.00 ± 3.26	47.60 ± 7.88
FRP-2	68.71	75.99	90.42	3.25	1.63 ± 0.76	266.8 ± 7.05	17.36 ± 2.40	23.86 ± 8.71

^a^ Young’s modulus, ^b^ ultimate tensile strength, ^c^ elongation at break, ^d^ modulus of toughness.

**Table 3:** The structure and mechanical properties of polyHIPE membranes with different molar ratios.

Sample	BA/St/DVB	E ^a^ (MPa)	σ ^b^ (kPa)	ε ^c^ (%)	M_T_ ^d^ (kJ·m^−3^)	BET (m^2^·g^−1^)	P_A_ (%)	P_T_ (%)	P_C_ (%)
RAFT-2	9/2/1	2.04 ± 0.34	347 ± 4.31	24.00 ± 3.26	47.60 ± 7.88	3.42	67.21	75.69	88.80
RAFT-3	7/4/1	10.09 ± 1.82	819.62 ± 33.31	17.50 ± 3.90	93.04 ± 12.28	3.85	69.79	75.57	92.35
RAFT-4	5/6/1	51.10 ± 2.68	664.4 ± 41.37	1.60 ± 0.66	6.24 ± 3.45	5.19	76.65	75.71	100 ^e^

^a^ Young’s modulus, ^b^ ultimate tensile strength, ^c^ elongation at break, ^d^ modulus of toughness, ^e^ Deviation in the theoretical calculation, P_C_ is recorded as 100% when P_A_ is greater than P_T_.

## Data Availability

The original contributions presented in this study are included in the article. Further inquiries can be directed to the corresponding author.

## References

[1] Favre E. (2022). The Future of Membrane Separation Processes: A Prospective Analysis. Front. Chem. Eng..

[2] DuChanois R.M., Porter C.J., Violet C., Verduzco R., Elimelech M. (2021). Membrane Materials for Selective Ion Separations at the Water–Energy Nexus. Adv. Mater..

[3] Aristizábal S.L., Lively R.P., Nunes S.P. (2023). Solvent and Thermally Stable Polymeric Membranes for Liquid Molecular Separations: Recent Advances, Challenges, and Perspectives. J. Membr. Sci..

[4] Song Z., Ma Y., Morrin A., Ding C., Luo X. (2021). Preparation and Electrochemical Sensing Application of Porous Conducting Polymers. TrAC Trends Anal. Chem..

[5] Li J., Li N., Zheng Y., Lou D., Jiang Y., Jiang J., Xu Q., Yang J., Sun Y., Pan C. (2022). Interfacially Locked Metal Aerogel inside Porous Polymer Composite for Sensitive and Durable Flexible Piezoresistive Sensors. Adv. Sci..

[6] Zheng B., Lin X., Zhang X., Wu D., Matyjaszewski K. (2020). Emerging Functional Porous Polymeric and Carbonaceous Materials for Environmental Treatment and Energy Storage. Adv. Funct. Mater..

[7] Shi J., Qin M., Aftab W., Zou R. (2021). Flexible Phase Change Materials for Thermal Energy Storage. Energy Storage Mater..

[8] Düerkop D., Widdecke H., Schilde C., Kunz U., Schmiemann A. (2021). Polymer Membranes for All-Vanadium Redox Flow Batteries: A Review. Membranes.

[9] Chen W., Xiang Y., Kong X.-Y., Wen L. (2022). Polymer-Based Membranes for Promoting Osmotic Energy Conversion. Giant.

[10] Yue H., Yuan L., Zhang W., Zhang S., Wei W., Ma G. (2018). Macrophage Responses to the Physical Burden of Cell-Sized Particles. J. Mater. Chem. B.

[11] Stucki M., Loepfe M., Stark W.J. (2018). Porous Polymer Membranes by Hard Templating—A Review. Adv. Eng. Mater..

[12] Liu Q., Xiong J., Lin W., Liu J., Wan Y., Guo C.F., Wang Q., Liu Z. (2025). Porous Polymers: Structure, Fabrication and Application. Mater. Horiz..

[13] Kovačič S., Žagar E., Slugovc C. (2019). Strength versus Toughness of Emulsion Templated Poly(Dicyclopentadiene) Foams. Polymer.

[14] Ronan W., Deshpande V.S., Fleck N.A. (2016). The Tensile Ductility of Cellular Solids: The Role of Imperfections. Int. J. Solids Struct..

[15] Kovačič S., Krajnc P., Slugovc C. (2010). Inherently Reactive polyHIPE Material from Dicyclopentadiene. Chem. Commun..

[16] Kovačič S., Preishuber-Pflügl F., Slugovc C. (2014). Macroporous Polyolefin Membranes from Dicyclopentadiene High Internal Phase Emulsions: Preparation and Morphology Tuning. Macromol. Mater. Eng..

[17] Kovačič S., Jeřábek K., Krajnc P., Slugovc C. (2012). Ring Opening Metathesispolymerisation of Emulsion Templated Dicyclopentadiene Giving Open Porous Materials with Excellent Mechanical Properties. Polym. Chem..

[18] Cameron N.R., Sherrington D.C. (1997). Preparation and Glass Transition Temperatures of Elastomeric PolyHIPE Materials. J. Mater. Chem..

[19] Carnachan R.J., Bokhari M., Przyborski S.A., Cameron N.R. (2006). Tailoring the Morphology of Emulsion-Templated Porous Polymers. Soft Matter.

[20] Pulko I., Smrekar V., Podgornik A., Krajnc P. (2011). Emulsion Templated Open Porous Membranes for Protein Purification. J. Chromatogr. A.

[21] Patten T.E., Xia J., Abernathy T., Matyjaszewski K. (1996). Polymers with Very Low Polydispersities from Atom Transfer Radical Polymerization. Science.

[22] Chiefari J., Chong Y.K., Ercole F., Krstina J., Jeffery J., Le T.P.T., Mayadunne R.T.A., Meijs G.F., Moad C.L., Moad G. (1998). Living Free-Radical Polymerization by Reversible Addition−Fragmentation Chain Transfer:  The RAFT Process. Macromolecules.

[23] Nothling M.D., Fu Q., Reyhani A., Allison-Logan S., Jung K., Zhu J., Kamigaito M., Boyer C., Qiao G.G. (2020). Progress and Perspectives Beyond Traditional RAFT Polymerization. Adv. Sci..

[24] Silverstein M.S. (2014). PolyHIPEs: Recent Advances in Emulsion-Templated Porous Polymers. Prog. Polym. Sci..

[25] Lubomirsky E., Khodabandeh A., Hofe T., Susewind M., Preis J., Hilder E.F., Arrua R.D. (2024). Single-Step Synthesis of Methacrylate Monoliths with Well-Defined Mesopores. Polymer.

[26] Li K., Zhang Q., Li J., Dai H., Zhou D. (2024). Dual-Templating Synthesis of Superhydrophobic Fe3O4/PSt Porous Composites for Highly Efficient Oil-Water Separation. J. Mol. Liq..

[27] Ferguson C.J., Hughes R.J., Nguyen D., Pham B.T.T., Gilbert R.G., Serelis A.K., Such C.H., Hawkett B.S. (2005). Ab Initio Emulsion Polymerization by RAFT-Controlled Self-Assembly. Macromolecules.

[28] (2006). Determination of Tensile Properties of Plastics Part 2: Test Conditions for Molded and Extruded Plastics.

[29] Yadav A., Ghosh S., Samanta A., Pal J., Srivastava R.K. (2022). Emulsion Templated Scaffolds of Poly(ε-Caprolactone)—A Review. Chem. Commun..

[30] Aronson M.P., Petko M.F. (1993). Highly Concentrated Water-in-Oil Emulsions: Influence of Electrolyte on Their Properties and Stability. J. Colloid Interface Sci..

[31] Foudazi R. (2021). HIPEs to PolyHIPEs. React. Funct. Polym..

[32] van Krevelen D.W., Te Nienhuis K. (2010). Properties of Polymers.

[33] Zhang F., Wang C., Huang X., Dong X., Chi H., Xu K., Bai Y., Wang P. (2024). New Approach for Preparation of Porous Polymers with Reversible Pore Structures for a Highly Safe Smart Supercapacitor. ACS Appl. Mater. Interfaces.

[34] Koler A., Brus J., Krajnc P. (2023). RAFT Polymerisation and Hypercrosslinking Improve Crosslink Homogeneity and Surface Area of Styrene Based PolyHIPEs. Polymers.

[35] Yu Q., Xu S., Zhang H., Ding Y., Zhu S. (2009). Comparison of Reaction Kinetics and Gelation Behaviors in Atom Transfer, Reversible Addition–Fragmentation Chain Transfer and Conventional Free Radical Copolymerization of Oligo(Ethylene Glycol) Methyl Ether Methacrylate and Oligo(Ethylene Glycol) Dimethacrylate. Polymer.

[36] Luo Y., Wang A.-N., Gao X. (2012). Pushing the Mechanical Strength of PolyHIPEs up to the Theoretical Limit through Living Radical Polymerization. Soft Matter.

